# Exploration of the Preventive and Therapeutic Effects of D-Lactate Administration in a Mouse MCAO Model

**DOI:** 10.3390/ph19030410

**Published:** 2026-03-02

**Authors:** Seyedeh Maryam Mousavi, Lara Buscemi, Julia Castillo-González, Melanie Price, Lorenz Hirt

**Affiliations:** 1 Stroke Laboratory, Neurology Service, Department of Clinical Neurosciences, Lausanne University Hospital Centre and University of Lausanne, CH-1011 Lausanne, Switzerland; seyedeh-maryam.mousavi@unil.ch (S.M.M.); lara.buscemiestefanell@unil.ch (L.B.); julia.castillogonzalez@unil.ch (J.C.-G.); write.lines.editing@gmail.com (M.P.); 2 Department of Fundamental Neurosciences, University of Lausanne, CH-1005 Lausanne, Switzerland

**Keywords:** ischemic stroke, D-lactate, blood–brain barrier breakdown, neuroprotection

## Abstract

**Background**: Stroke is a major global risk to human health due to its high incidence, mortality, and prevalence of associated long-term disabilities. Recent studies have highlighted a significant impact of the gut–brain axis and metabolites derived from intestinal microbiota on modulating neurological disorders, including stroke. **Methods**: In this study, we investigated the effects of pre- and post-treatment with D-lactate, a lactate stereoisomer mainly produced by certain gut bacteria, on stroke outcome using a transient middle cerebral artery occlusion (MCAO) mouse model. For this purpose, male C57BL/6J mice received a single administration of D-lactate or vehicle (PBS) via the tail vein either before the MCAO surgery, as a preventive approach, or upon reperfusion, as a therapeutic paradigm. Functional outcome was assessed daily using a standard neuroscore and the adhesive removal test until day three post-surgery, when mice were sacrificed. **Results**: Our results indicated no significant difference in infarct size, measured using cresyl violet staining, between the D-lactate and PBS groups in both pre- and post-treatment experiments. In addition, evaluation of neurological deficits and sensorimotor function showed no statistically significant differences between the interventions throughout the experiment. **Conclusions**: The present data suggest that treatment with D-lactate does not show a beneficial effect in our C57BL/6J mouse MCAO model.

## 1. Introduction

Stroke is one of the leading causes of mortality and the predominant cause of long-term disability worldwide, with ischemic stroke accounting for 87% of all cases [1]. Despite the recent advances in thrombolysis and endovascular thrombectomy, not all patients are eligible for, or can fully benefit from, the only clinically approved therapeutic options in the acute phase [2]. Therefore, there is a pressing need to identify novel strategies that could potentially improve the management of stroke.

Recent evidence has highlighted the importance of communication between the gut and the brain in regulating mental and neurological disorders, including stroke, indicating the significant role played by the gut microbiota and microbial-derived metabolites. This complex bidirectional crosstalk, also known as the gut–microbiota–brain axis, is mediated by the immune, nervous, and endocrine systems [3,4]. Stroke substantially alters the composition of the intestinal flora in a stroke severity-dependent fashion and additionally affects gut motility and gut epithelial barrier permeability [5,6,7]. Alongside intestinal perturbations, a drastic disruption of the structural composition and function of the blood–brain barrier (BBB) occurs after the ischemic event. The BBB serves as a selective barrier protecting the brain microenvironment, and when compromised after stroke, it leads to increased permeability, allowing the passage of toxins, pathogens, and immune cells into the brain, further causing edema and inflammation [8,9]. Emerging evidence relates BBB dysfunction to the composition and state of the gut microbiota, with cytokines, systemic inflammation, bacterial products, and metabolites serving as the primary mediators of this interaction [8,9].

D-lactate, the D-enantiomer of lactate, is predominantly produced by certain bacteria inside the gastrointestinal tract, including *Lactobacillus* species, with only minimal amounts formed by mammalian metabolic pathways [10]. Several *in vivo* and *in vitro* studies have suggested that D-lactate is mainly non-metabolized compared to L-lactate in cerebral tissue [11,12,13]. However, it can still act as a partial agonist of the hydroxycarboxylic acid receptor 1 (HCAR1) [14]. Some previous research has pointed towards no effect or adverse effects of D-lactate on brain function [15]. In contrast, in 2015, a study from our group demonstrated a beneficial effect of D-lactate administration in *in vivo* (ICR-CD1 outbred mice) and *in vitro* models of ischemia, reducing the infarct volume and improving the neurological score and reducing cell death, respectively [14].

The present study aimed to re-examine and improve our understanding of the effects of exogenous administration of the gut metabolite D-lactate in the acute phase after stroke. We investigated D-lactate administration both before ischemia and at reperfusion in the context of the BiotaBB project, focusing on the effect of microbial gut metabolites on blood–brain barrier integrity in stroke. The study evaluated the influence of D-lactate on the ischemic brain lesion, functional outcome, and BBB permeability.

## 2. Results

### 2.1. Pretreatment with D-Lactate Before Stroke Did Not Reduce Infarct Size or Attenuate the Increased BBB Permeability

**Figure 1:**
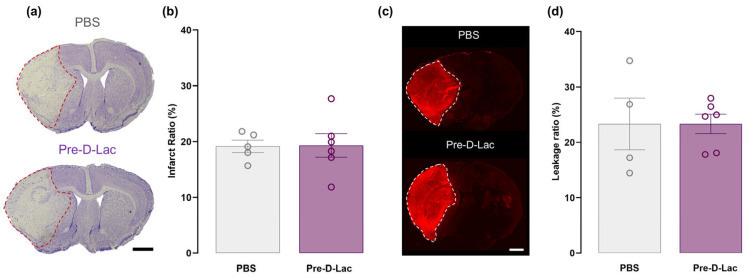
Evaluation of infarct ratio and BBB permeability after pretreatment with D-lactate. (**a**) Example of a coronal brain section from each group stained with cresyl violet. Dashed lines delineate the ischemic lesion. Scale bar: 1 mm. (**b**) The infarct ratio was measured as the percentage of ischemic lesion area relative to the whole brain area (PBS; n = 5, Pre-D-Lac: n = 6). (**c**) Representative immunofluorescence images from each group showing endogenous IgG extravasation with white dashed lines delineating the leakage area. Scale bar: 1 mm. (**d**) The ratio of IgG leakage into the brain parenchyma was measured as the percentage of IgG-stained area relative to the brain area (PBS; n = 4, Pre-D-Lac: n = 6). Data are shown as mean ± SEM with each open circle representing one animal. Unpaired *t*-test.

D-lactate at the dose of 1 mmol/kg or an equivalent volume of PBS was administered via the tail vein one hour before the MCAO surgery in the pretreatment experiment, and animals were euthanized three days later. The extent of the ischemic damage was assessed on cresyl violet-stained consecutive coronal brain sections. We observed that at three days after the MCAO surgery, the percentage of infarcted tissue was similar between the D-lactate and the PBS groups (Figure 1a,b).

We evaluated the level of ischemia-induced BBB breakdown by assessing the extravasation of endogenous immunoglobulin G (IgG) into the brain parenchyma at day 3 after stroke using immunofluorescence. Three days after MCAO, all animals exhibited extensive IgG leakage into the ipsilateral hemisphere (Figure 1c). No substantial difference in the IgG infiltration ratio was found between the intervention groups (Figure 1c,d), indicating that D-lactate administration prior to surgery did not protect against increased stroke-induced BBB permeability.

### 2.2. Pretreatment with D-Lactate Did Not Improve the Neurological Deficit or Sensorimotor Function

**Figure 2:**
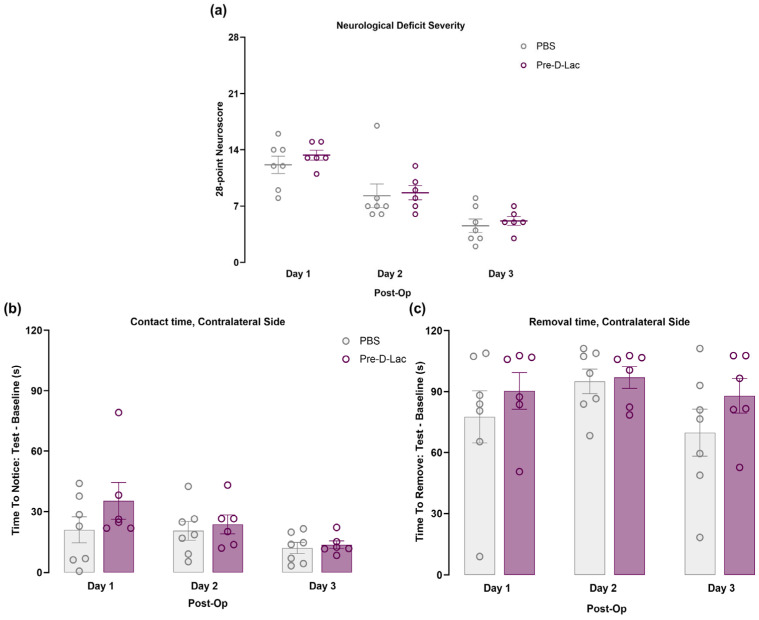
Assessment of functional outcome following pretreatment with D-lactate. (**a**) Neurological function was evaluated daily using the 28-point neuroscore, where 28 represents the maximum deficit, and 0 shows no deficit, over the 3 days of the experiment. (**b**,**c**) The adhesive removal test was conducted one day before the surgery (baseline), and every day after MCAO to assess (**b**) time to contact the tape placed on the functionally impaired right forepaw, and (**c**) time to remove this tape (PBS; n = 7, Pre-D-Lac: n = 6). Results are illustrated as mean ± SEM, with each open circle representing one mouse. Two-way ANOVA followed by Sidak’s post hoc test. Post-Op indicates days after surgery.

To assess functional outcome, neurological deficit was evaluated daily until sacrifice using a 28-point neuroscore. All animals demonstrated functional recovery throughout the 3-day experiment. Preadministration of D-lactate, however, did not lead to better outcomes with time compared to the PBS group (Figure 2a). Additionally, we performed the adhesive tape removal test, which allows assessing fine sensorimotor function involving the mouth and forelimb digits. We observed that the mean times required to contact or to remove the tape were longer in both groups on days 1, 2, and 3 following stroke compared with baselines. However, we did not find significant differences in these measures between the two groups or across time points (Figure 2b,c).

### 2.3. Administration of D-Lactate at Reperfusion Did Not Significantly Affect the Infarct Volume or Ameliorate BBB Leakage

**Figure 3:**
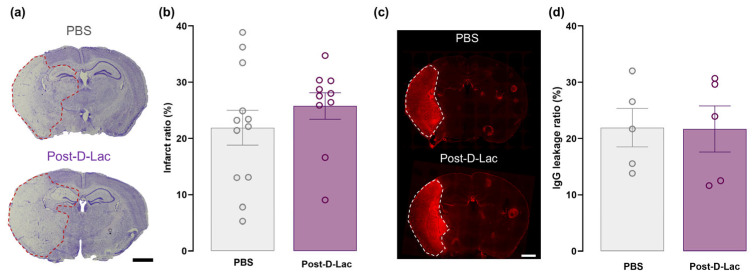
Evaluation of the lesion extension and BBB breakdown following D-lactate treatment at reperfusion. (**a**) Representative image of cresyl violet-stained brain coronal sections from a PBS (**top**) and a D-lactate animal (**bottom**) injected at reperfusion, with dashed red lines outlining the lesion area. Scale bar: 1 mm. (**b**) Quantification of total infarct area expressed as a percentage of the total area of mice injected with PBS (n = 12) or D-lactate (n = 10) at reperfusion. (**c**) Representative images of endogenous IgG infiltration from blood into the brain parenchyma from a PBS- and a D-lactate-injected mouse at reperfusion, with white dashed lines outlining the extravasation area. (**d**) Quantification of the IgG leakage area expressed as a percentage of the total area of PBS- (**top**) and D-lactate-injected mice (PBS, n = 5; Post-D-Lac, n = 5). Scale bar: 1 mm. Findings are shown as mean ± SEM, with each open circle representing one animal. Unpaired *t*-test.

We next evaluated the efficacy of D-lactate when administered at the onset of reperfusion (post-stroke treatment), a paradigm with greater clinical translational potential. Our findings revealed that post-stroke D-lactate administration did not have a significant effect on the infarct ratio compared to PBS-injected animals (Figure 3a,b). Individual brain region analysis showed that post-treatment with D-lactate did not significantly affect infarct volume in any of the analysed brain regions (Figure S1a) or across individual coronal sections (Figure S1b).

Consistent with the infarct ratio data, IgG extravasation analysis showed no statistically significant difference between animals injected with PBS at reperfusion and those receiving D-lactate. Both experimental groups displayed a similar pattern of parenchymal IgG accumulation within the ipsilateral hemisphere (Figure 3c,d).

### 2.4. Functional Recovery of Animals Following MCAO Was Not Improved by Treatment with D-Lactate Post-Stroke

**Figure 4:**
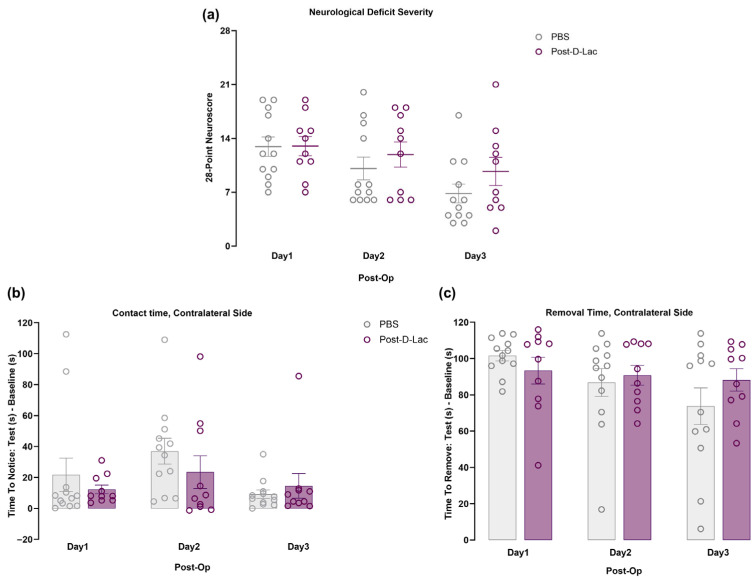
Assessment of functional outcome following D-lactate post-treatment. (**a**) Time course analysis of the functional outcome after MCAO using the 28-point neuroscore in PBS and D-lactate post-treated animals. (**b**,**c**) Adhesive removal test. (**b**) Time to contact and (**c**) time to remove the tape placed on the contralateral forepaw were compared between the PBS and D-lactate treated animals (PBS: n = 12; Post-D-Lac: n = 10). Data are shown as mean ± SEM, with each dot representing one animal. Two-way ANOVA with Sidak’s multiple comparisons test. Post-Op refers to days after surgery.

Similar to the pretreatment study, the majority of animals administered PBS or D-lactate at reperfusion exhibited recovery over time. However, no statistically significant difference in the 28-point neuroscore was observed between the two treatments (Figure 4a). Additionally, sensorimotor function was evaluated using the adhesive removal test. Similar to the pretreatment paradigm, no statistically significant differences were noted for adhesive tape contact or removal times between the two groups at the different time points (Figure 4b,c).

## 3. Discussion

We designed the current study within the ERA-NET NEURON “BiotaBB” consortium, the objective being to investigate the effects of microbiota-derived metabolites on the gut–brain axis and blood–brain barrier (BBB) integrity and to expand upon previous findings from our group suggesting that D-lactate shows neuroprotective characteristics when administered after stroke modelled by MCAO in outbred ICR-CD1 mice or after oxygen–glucose deprivation *in vitro* [14]. However, our present blinded and randomized investigation revealed that the intravenous injection of D-lactate, administered either 1 h prior to ischemia induction or at the time of reperfusion, did not significantly decrease infarct size, improve neurological function, or ameliorate sensorimotor deficits in the experimental C57BL/6J mouse stroke model used. Moreover, D-lactate treatment did not reduce the extravasation of plasma proteins into brain parenchyma in either treatment paradigm. The current findings contradict our initial hypothesis and the earlier results of a beneficial effect of D-lactate *in vivo*. On the other hand, they agree with observations showing no therapeutic effect of D-lactate in an *in vitro* model of the ischemic penumbra [16], and its detrimental impact when injected before MCAO induction in a mouse MCAO model [17].

The divergence between our present results and prior findings could be ascribed to several experimental variables. Although we chose to use the same dose of 1 mmol/kg body weight as previously used for D- [14] and L-lactate experiments [18], our MCAO model differs from the earlier D-lactate study in several aspects. In the present model, a filament was inserted through the external carotid artery (ECA) to facilitate reperfusion. Additionally, we used a different mouse strain, modified the ischemia duration, and adjusted the time of sacrifice. Specifically, the occlusion time was reduced from 45 min to 25 min to improve survival, as the animals were maintained for 3 days post-MCAO instead of 2 days. These changes may account for the differences in measured outcomes. A compelling argument comes from the severely restricted metabolism of D-lactate in the mammalian system. While once considered entirely non-metabolizable, in 2015, Castillo et al. demonstrated that D-lactate can be oxidized by the rodent brain, indicating the presence and activity of D-lactate dehydrogenase (D-LDH), which is expressed at markedly lower levels in brain tissue than L-lactate dehydrogenase (L-LDH) [14]. It is possible that, in our refined MCAO model, using a different strain of mice, the C57BL/6J versus ICR-CD1 strain, the D-LDH expression pattern or metabolic profile that influences D-lactate consumption after ischemia are distinct. In addition, despite testing two experimental paradigms, namely pretreatment as a preventive strategy and post-treatment as a therapeutic approach, the temporal time window and dose of D-lactate for this model may not have been optimal. Beyond methodological considerations, mechanistic factors may also contribute to the observed lack of effect. In the present study, we did not assess D-lactate binding to the HCAR1. Nevertheless, prior work from our group suggested that HCAR1 is unlikely to be the primary mechanism of action in neuroprotection as selective pharmacological activation of HCAR1 failed to mimic L-lactate-mediated protection [19], and genetic deletion of HCAR1 reduced ischemic damage and improved behavioural outcomes compared with wild-type mice following MCAO [20]. Given these findings, together with the absence of detectable benefit from D-lactate and its lower affinity for HCAR1 relative to L-lactate, we did not pursue additional receptor-level investigations. Our conclusions are therefore limited to a lack of D-lactate effect under the present experimental conditions and do not exclude potential strain-specific differences in HCAR1 signaling or D-lactate metabolism between the two mouse strains used in the two studies.

While L-lactate is recognized as a significant alternative energy source in the brain with neuroprotective potential [19], the limited literature on D-lactate in the context of neurological disorders shows mixed findings. Under normal physiological conditions, L-lactate is the predominant enantiomer in mammals, with D-lactate produced in minimal quantities. In situations where there is excessive D-lactate buildup, its accumulation can lead to D-lactic acidosis and secondary injury, as mammalian lactate dehydrogenase has a much higher affinity for L-lactate compared to its D-enantiomer [20,21]. Notably, increased D-lactate plasma levels were reported in ischemic stroke patients [22]. D-lactic acidosis primarily links excessive concentrations of D-lactate to neurological symptoms such as mental state changes, dysarthria, disorientation, gait disturbance, ataxia, and nystagmus [23,24]. These neurological changes have also been shown to correlate with D-lactate changes in the cerebrospinal fluid of animals in experimental studies [20]. Another study has shown that intracranial injection of D-lactate impairs the neonatal chick memory process, which is explained by the inhibition of L-lactate uptake into neurons and interference with astrocytic metabolism [25,26]. Various studies have further assessed the influence of D-lactate on memory and learning, indicating that its effects may be inhibitory [25,26], neutral [27], or beneficial [28], contingent upon the concentration and timing of administration. In the context of Alzheimer’s disease, research indicates that elevated D-lactate levels are associated with exacerbated mitochondrial dysfunction, neuronal injury, and neuroinflammation [29,30]. Furthermore, as a result of disruption of the gut epithelium barrier following traumatic brain injury, D-lactate is considered a blood biomarker for injury severity [31,32]. Collectively, these complementary results highlight the multifaceted and complex influence of excessive D-lactate levels in neurological disorders, indicating a dual role of this microbiota-derived metabolite as both a neurotoxic and neuroprotective agent.

More broadly, our findings reflect a persistent challenge in preclinical stroke research. Despite significant advancements in experimental stroke models, only a limited number of promising preclinical candidates demonstrating efficacy in animal studies have progressed to clinical trials, to end being proven ineffective [33,34]. Systematic reviews have identified multiple sources of bias that compromise the reproducibility and translational efficacy of preclinical research, including insufficient statistical power, lack of randomization and blinding, and publication bias favoring positive results [33]. Biomedical research prioritizes creativity above validation, cultivating an environment where negative or contradictory findings face barriers to publication, despite their importance in preventing the pursuit of ineffective approaches [35]. The current study refined the experimental design through modifying the surgical approach, employing two experimental paradigms, and integrating blinding and randomization to guarantee unbiased outcome assessment. Our results do not suggest unsuccessful outcomes; instead, they demonstrate the need for careful confirmatory efforts, essential to validate initial positive experimental data prior to clinical use.

## 4. Materials and Methods

### 4.1. Middle Cerebral Artery Occlusion (MCAO)

A total number of 49 male C57BL/6J mice (Charles River, L’Arbresle, France) aged 8 to 12 weeks were housed under standard environmental conditions with a 12 h/12 h light/dark cycle, regulated humidity and temperature, and *ad libitum* access to food and water. We conducted all experimental procedures in accordance with Swiss laws for the protection of animals with the approval of the Vaud Cantonal Veterinary Office under the animal experimentation authorization license VD2017.7c (7 July 2023). All the inclusion and exclusion criteria, including regional cerebral blood flow (rCBF) parameters, humane endpoints, and successful intravenous injection, were predefined to ensure compliance with the ARRIVE guidelines. Specifically, we included adult male C57BL/6J mice in our study. Mice were excluded if they did not satisfy pre-established rCBF criteria (rCBF below 20% of the initial value during occlusion time and rCBF above 50% of the baseline value within 10 min of filament removal) or if the intravenous injection failed. They were also excluded if they needed to be sacrificed for humane reasons. In accordance with our animal experimentation authorization license, a well-being score sheet, with a score ranging from 0 (normal) to 3 (severe deviation from normal), was employed to assess the mice daily. We examined their physical condition, posture, and spontaneous activity, neurological deficits, and epileptic activity, as well as body condition and dehydration. A humane endpoint was reached when any individual score obtained 3 points, and the animal was therefore euthanized (see Tables S1 and S2). Sample size was determined by prior power analysis based on our previous publications and the approved animal experimentation license: A mean lesion volume reduction of 40 mm^3^ to 25 mm^3^ (SD 10 mm^3^) could be detected with a power of 80%, a significance of 0.05 at N = 7 per group.

Mice underwent the MCAO surgery using the intraluminal filament method as described previously [36]. Surgeries were carried out under isoflurane anesthesia (1.5–2% in a mixture of 70% N_2_O and 30% O_2_) with constant monitoring of regional cerebral blood flow (rCBF) using laser-Doppler flowmetry (Perimed AB, Järfälla, Sweden) through a flexible probe fixed to the skull at 1 mm posterior and 6 mm lateral from the bregma. Transient focal cerebral ischemia was induced on the left side by inserting a silicon-coated monofilament (602212PK10Re, Doccol Corp, Sharon, MA, USA) into the external carotid artery and advancing it through the internal carotid artery until it occluded the origin of the middle cerebral artery. The filament was withdrawn after 25 min of occlusion, followed by the removal of the ligation on the common carotid artery to facilitate reperfusion. Surgeries were considered successful if rCBF was below 20% of the initial value during occlusion time and reached a reperfusion rate above 50% of the baseline value within 10 min of filament removal. Seventy-two hours post-ischemia, the surviving animals were euthanized by an intraperitoneal injection of a lethal dose (150 mg/kg) of sodium pentobarbital, followed by intracardiac perfusion with 4% paraformaldehyde diluted in PBS at pH 7.4. Dissected brains were post-fixed overnight at 4 °C, cryoprotected in 30% sucrose for a minimum of 48 h, and subsequently cryosectioned into 25 μm coronal slices.

### 4.2. Drug Administration

Single tail vein injections of either vehicle (PBS, 5 μL/g, pH 7.4) or sodium D-lactate (Merck & Cie, Buchs, Switzerland; Cat. No. 71716, 1 mmol/kg body weight, based on the dose used in [14]) at pH 7.4 were administered to mice in a randomized and blinded approach. Two independent experiments were conducted: (1) a pre-D-lactate experiment, which involved the injection of D-lactate one hour before the start of the MCAO surgery, and (2) a post-D-lactate experiment, which involved the injection at the time of reperfusion.

### 4.3. Infarct Size Quantification

Twelve 25 μm thick coronal brain slices, spaced 700 μm apart, were obtained from each animal. Sections were stained with cresyl violet [18], imaged under a light stereomicroscope (Nikon SMZ25, Nikon Corporation, Tokyo, Japan) at a magnification of 1.2×, and analysed using Fiji software (version 2.14.0/1.54 f). The infarct ratio was measured by dividing the sum of the infarct areas by the total brain area and multiplying by 100. To further analyse the extent of damage in the post-D-lactate experiment, the total damaged area on each coronal section was quantified and compared to the PBS group. Additionally, these values were multiplied by the intersectional distance to calculate the infarct volume in each brain structure in both groups.

### 4.4. Assessment of Functional Deficits

#### 4.4.1. 28-Point Neuroscore

The 28-point neuroscore is a standard test ranging from 0 (no deficit) to 28 (terminal endpoint reached) that evaluates body symmetry, gait, climbing, spontaneous circling behaviour, forelimb symmetry, compulsory circling, and whisker response [37]. The test was conducted daily after MCAO until the end of the experiment at day three.

#### 4.4.2. Adhesive Removal Test

We conducted the adhesive tape removal test based on a previously tested protocol [38]. Adhesive tape was cut into rectangular pieces measuring 3 × 4 mm. At the beginning of the test, mice were placed in a transparent cylinder for a habituation period of 60 s. Then, a prepared adhesive tape was firmly positioned underneath each forepaw. Mice were immediately returned to the cylinder and recorded for 120 s. Each mouse had three consecutive trials per day. The time to contact and the time to remove each adhesive tape were noted by an experimenter blinded to the treatment. We considered contact as the time when the paw was either shaken or touched with the muzzle, showing contact with the tape. The removal time was when the mouse completely removed the tape from each forepaw. The test was performed one day before the MCAO surgery to obtain baseline reference values and each day after surgery until the sacrifice. The results are expressed as the average of the three daily trials relative to the respective baseline.

### 4.5. Evaluation of Blood–Brain Barrier Permeability

To evaluate the integrity of the BBB, immunofluorescence labeling of extravasated IgG was done as previously described [39]. In brief, a series of 25 μm brain sections were washed with PBS, then blocked and permeabilized with PBS containing 1% BSA, 0.1% Triton X-100, and 10% horse serum for 60 min at room temperature. Next, slides were incubated overnight at 4 °C with an Alexa Fluor 594-conjugated goat anti-mouse IgG (Invitrogen, Cat. No. A11032) in PBS containing 1% BSA, 0.1% Triton X-100, and 2% horse serum. Images of whole sections were captured using a Zeiss Z1 slide scanner (Carl Zeiss AG, Oberkochen, Germany), and the ratio of IgG leakage was quantified using QuPath software (version 0.5.1).

### 4.6. Statistical Analysis

Data are presented as mean ± standard error of the mean (SEM). Statistical analyses were performed using GraphPad Prism (version 8.3.0). Comparisons between two groups were conducted using an unpaired Student’s *t*-test for normally distributed data or the Mann–Whitney *U* test for non-normally distributed data. A two-way repeated-measures analysis of variance (ANOVA) was used to assess the effects of treatment and time, followed by Sidak’s post hoc test for multiple comparisons. A *p*-value < 0.05 was considered statistically significant.

## Data Availability

The original contributions presented in this study are included in the article. Further inquiries can be directed to the corresponding author.

## Supplementary Materials

The following supporting information can be downloaded at: https://www.mdpi.com/article/10.3390/ph19030410/s1. Figure S1. Extended cresyl violet staining assessment of infarct size following D-lactate post-treatment. Table S1. Summary of the animals used in the pretreatment with D-lactate experiment. Table S2. Summary of the animals used in the post-treatment with D-lactate experiment.

## Abbreviations

The following abbreviations are used in this manuscript:

MCAO	Middle cerebral artery occlusion
PBS	Phosphate-buffered saline
BBB	Blood–brain barrier
HCAR1	Hydroxycarboxylic acid receptor 1
IgG	Immunoglobulin G
ECA	External carotid artery
D-LDH	D-lactate dehydrogenase
rCBF	Regional cerebral blood flow
SD	Standard deviation
SEM	Standard error of mean
ANOVA	Analysis of variance
