# Supplementary material for: Exploration of the Preventive and Therapeutic Effects of D-Lactate Administration in a Mouse MCAO Model
Source: Pharmaceuticals (Basel). 2026 Mar 2;19(3):410. doi: 10.3390/ph19030410 (PMC13029295; doi:10.3390/ph19030410)
Supplement: Supplementary file 1 [file pharmaceuticals-19-00410-s001.zip › pharmaceuticals-4153559-supplementary.pdf]

*Article*

# Exploration of the Preventive and Therapeutic Effects of D-Lactate Administration in a Mouse MCAO Model

**Seyedeh Maryam Mousavi [1,2], Lara Buscemi [1,2], Julia Castillo-González [1,2], Melanie Price [1,2] and Lorenz Hirt [1,2,*]**

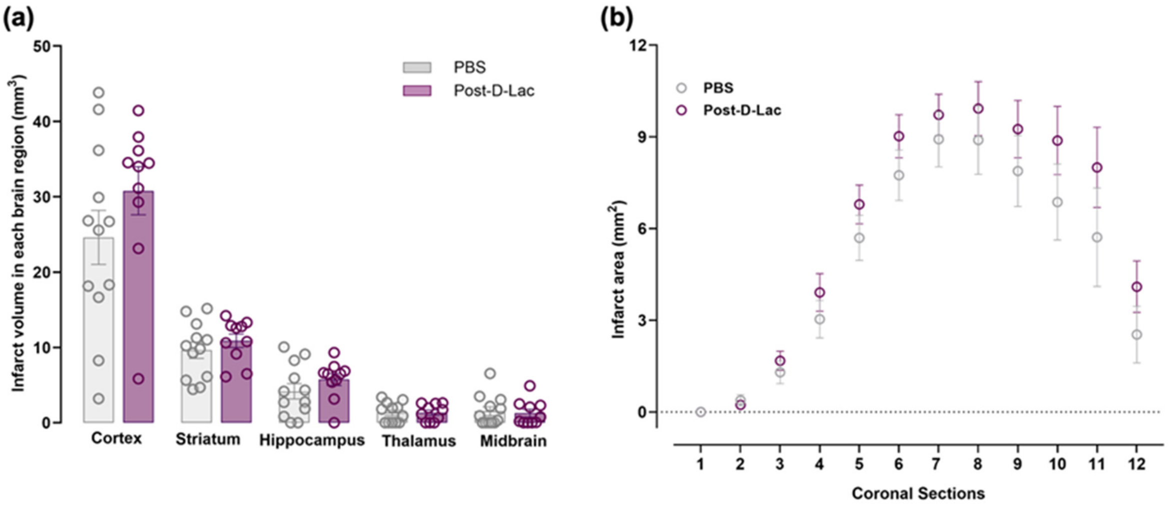


**Figure S1.** Extended cresyl violet staining assessment of infarct size following D-lactate post-treatment. (a) Regional evaluation of infarct size across cerebral areas illustrates the distribution of ischemic damage from anterior to posterior regions in both the PBS and post-lactate treatment groups. Multiple unpaired t-test. Each dot corresponds to one animal. (b) Quantification of the injured area in each coronal section along the rostro-caudal axis between the D-lactate and PBS-treated animals. Two-way ANOVA with Sidak's multiple comparisons test. Data are shown as mean ± SEM.

**Table S1.** Summary of the animals used in the pretreatment with D-lactate experiment.

| | PBS | Pre-D-lactate |
|---|---|---|
| Total | 10 | 10 |
| Humane endpoint | 1 | 2 |
| Completed | 7 | 6 |
| Infarct size study | 5 | 6 |
| Behavioural study | 7 | 6 |

Twenty mice were utilized in the pretreatment experiment, with 10 in the PBS group and 10 in the D-lactate group. Due to its poor overall condition, we euthanised one PBS-treated mouse at 24 hours after MCAO, as well as two mice that received D-lactate at 24 and 48 hours following the surgery, respectively. Two animals from the PBS-injected group were eliminated because their cerebral

blood flow did not exceed 50% of the baseline value following filament removal, and one animal from the D-lactate group. Another animal was eliminated from the D-lactate group due to the unsuccessful intravenous injection. Only 5 of the 7 mice that finished the experiment qualified for infarct size measurement in the PBS group due to issues with brain tissue processing. All animals that completed the experiment were incorporated into the behavioural evaluation.

**Table S2.** Summary of the animals used in the post-treatment with D-lactate experiment.

| | **PBS** | **Post-D-lactate** |
|---|---|---|
| Total | 15 | 14 |
| Humane endpoint | 3 | 0 |
| Completed | 12 | 10 |
| Infarct size study | 12 | 10 |
| Behavioural study | 12 | 10 |

Twenty-nine animals were used in the post-treatment group, including 15 mice in the PBS-injected group and 14 in the D-lactate administered group. 3 PBS-injected mice reached the humane endpoint prior to the scheduled sacrifice and were euthanised. No animal in the D-lactate group reached the humane endpoint; however, four animals in this group were excluded from the data analysis due to insufficient cerebral blood reperfusion or unsuccessful intravenous treatment administration.
